# Inhalation delivery dramatically improves the efficacy of topotecan for the treatment of local and distant lung cancer

**DOI:** 10.1080/10717544.2021.1912209

**Published:** 2021-04-16

**Authors:** Philip J. Kuehl, Christin M. Yingling, Devon Dubose, Michael Burke, David A. Revelli, Wenshu Chen, Wendy W. Dye, Steven A. Belinsky, Mathewos Tessema

**Affiliations:** a Lung Cancer Program, Lovelace Respiratory Research Institute, Albuquerque, NM, USA; b Lonza-Bend Research Institute, Bend, OR, USA

**Keywords:** Topoisomerase inhibitor, chemotherapy, non-small cell lung cancer, inhalation therapy, dry powder, aerosol, KRAS, EGFR mutant

## Abstract

Topotecan is potent anti-cancer drug approved for various malignancies but hematopoietic toxicities undermine its wider application and use of its most effective dose. This study aims to improve these limitations through inhalation-delivery. The pharmacokinetics, efficacy, and toxicity of 2–5 times lower inhalation doses of topotecan dry-powder were compared with the standard intravenous (IV) delivery once/twice-a-week. Human-derived EGFR-mutant (H1975), KRAS-mutant (A549), and EGFR/KRAS wild-type (H358) orthotopic and distant lung tumors were evaluated in murine models. Inhalation of 1 mg/kg topotecan significantly improved the half-life and drug exposure (area under the curve, AUC) compared to 5 mg/kg via IV-delivery. AUCs (h*ng/mL) for inhaled/IV topotecan in plasma, lung, liver, and brain were, 831/888, 60,000/1080, 8380/4000, and 297/15, respectively; while the half-life was also greatly increased in these tissues. The average lung tumor burden of H358-derived tumors was reduced from 15.0 g to 8.4 g (44%) in rats treated once-a-week with 2 mg/kg IV and 1.8 g (88%) with 1 mg/kg inhaled topotecan, corroborating previous findings using A549- and H1975-derived orthotopic lung tumors. Importantly, inhaled topotecan showed superior efficacy in suppressing lung tumors at distant sites. The growth of H1975- and H358-derived subcutaneous xenografts were completely arrested and A549-derived tumors were significantly reduced in mice treated twice-a-week with 1 mg/kg inhaled topotecan compared to a minor (H1975 and H358) or no reduction (A549) with twice-a-week 5 mg/kg IV topotecan.

## 1. Introduction

The last two decades have seen important advances that improved the efficacy of cancer therapy. Non-small cell lung cancer (NSCLC) patients that account for approximately 85% of all lung cancer cases are among those who benefited the most from these advances. First targeted therapy using tyrosine kinase inhibitors (TKIs) and more recently immunotherapy using immune checkpoint inhibitors (ICIs) became the standard-of-care and improved survival of NSCLC patients (Paez et al., 2004; Tsao et al., 2005; Soda et al., 2007; Socinski et al., 2018; West et al., 2019). However, the benefits of TKIs and ICIs are limited to a small subset of patients whose tumors show specific sensitivity markers that activate cancer-driver tyrosine kinases such as EGFR, BRAF, and EML4-ALK mutations or translocations (Paez et al., 2004; Tsao et al., 2005; Soda et al., 2007) or over-expressing checkpoint proteins such as PD-1, PD-L1, and CTLA‐4 (Brahmer et al., 2012; Meng et al., 2017; Ribas & Wolchok, 2018). In addition, nearly all targeted therapy responsive NSCLC patients develop resistance within the first 2 years (Pao & Girard, 2011; Camidge et al., 2014) while efficacy of immunotherapy in some advanced NSCLC patients including those with PD-L1 positive tumors is not much better than standard chemotherapy (Carbone et al., 2017). As a result, even in this era of targeted- and immuno-therapy, chemotherapy remains a major treatment option for the majority of advanced stage lung cancer patients. It serves both as an alternative and/or complementary treatment option to TKIs and ICIs (Carbone et al., 2017; Meng et al., 2017; Gandhi et al., 2018; Wang et al., 2020). Therefore, improving the efficacy and toxicity of chemotherapy for lung cancer, which remains the leading cause of cancer‐related deaths worldwide, will play a major role in improving the survival of many of the 1.76 million people lung cancer kills annually (Bray et al., 2018; Siegel et al., 2020).

The discovery and development of a new anticancer agent is an extremely lengthy and costly process with high failure rates. The wider use of some potent anticancer drugs that pass these stringent developmental and approval processes is further undermined by severe toxicity in some patients. Thus, improving or re-purposing approved but rarely used potent anticancer drugs such as topotecan will accelerate the path toward wider clinical use and the potential to quickly serve lung cancer patients as an alternative or complementary drug to other chemo-, TKI-, and/or ICI- therapies. Topotecan is a topoisomerase-I inhibitor derived from camptothecin (CPT), a naturally occurring compound in the Chinese plant called *Camptotheca acuminate* (Venditto & Simanek, 2010). The US Food and Drug Administration (FDA) approved the intravenous (IV) formulation of topotecan (US brand name Hycamtin) for multiple cancer types in 1996 and its oral capsule formulation in 2007 (O'Brien et al., 2006, 2007; Eckardt et al., 2007). Although topotecan is mostly used in small cell lung cancer, clinical trials using it as a single-agent or in combination with other drugs have also demonstrated efficacy for NSCLC (Lynch et al., 1994; Perez-Soler et al., 1996; Kindler et al., 1998; Weitz et al., 2000; Ramlau et al., 2006; Jones et al., 2008). The anti-tumor activity of topotecan in advanced NSCLC patients is comparable to the standard second-line drugs such as paclitaxel and docetaxel (Ramlau et al., 2006; Jones et al., 2008). However, the wider clinical use of topotecan is greatly restricted and its efficacy undermined by severe dose-limiting hematological toxicities in some patients.

The purpose of this study was to investigate the potential of inhalation delivery to improve the efficacy and clinical use of topotecan for lung cancer therapy. We hypothesized that targeted delivery of topotecan into the lungs through inhalation will increase drug exposure of the primary tumor, its local metastases, and minimize the potential role of these local tumors as the main sources of distant metastasis. Since inhalation directly delivers topotecan into the lungs, lower doses of the drug will achieve much higher local concentration and efficacy than the standard IV delivery. In addition, the lower inhalation doses plus the relatively slower release from the lungs into the systemic circulation will significantly reduce the systemic maximum concentration (*C*_max_), exposure of remote tissues most vulnerable to its toxicities seen following IV delivery (e.g. hematopoietic tissues). This will improve lung cancer patients’ tolerance to topotecan therapy thereby expanding its clinical use. Furthermore, we hypothesized that the retention and slower release of inhaled topotecan from the lungs to the systemic circulation minimizes its rapid excretion, prolongs it half-life, augments exposure of distant tumors, and improves its efficacy against metastatic lung cancer. These hypotheses were tested using human derived EGFR-mutant (H1975), KRAS-mutant (A549), and EGFR/KRAS wild-type (H358) orthotopic and distant lung tumors in various murine models. KRAS and EGFR mutations are two of the most common drivers of lung cancer that are responsible for approximately 25 and 15% of all NSCLC cases (Pao & Girard, 2011; Martin et al., 2013). Thus, comparing the efficacy of inhaled topotecan to the standard IV delivery using these three lung cancer types in an orthotopic and distant lung tumor setting allowed us to clearly define its potential use for the vast majority of lung cancer patients. Finally, a pilot toxicity study was conducted to evaluate the cumulative toxicity of topotecan inhalation at doses that showed strong efficacy against local and distant lung tumors.

## 2. Materials and methods

### 2.1. Drugs, animals, and lung cancer cell lines used

The spray-dried powder formulation of topotecan was manufactured from (S)-topotecan (Toronto Research Chemicals, Inc., Toronto, Canada) and its physical, chemical, and aerosol characteristics were determined as described (Kuehl et al., 2018). The aqueous formulation of topotecan for IV delivery was prepared immediately prior to injection according to the recommendation for HYCAMTIN^®^ (topotecan) for injection. A total of 48 male 6–8 weeks old Rowett nude rats (Cr:NIH-rnu) for the orthotopic lung cancer study were obtained from Envigo (Indianapolis, IN). Sixty female, 6–8 weeks old, CD-1^®^ IGS mice for pharmacokinetics (PKs), 54 female, 6–8 weeks old athymic nude mice (Crl:NU(NCr)-Foxn1nu) for subcutaneous xenografts, and 12 male, 8 weeks old Sprague Dawley rats for a pilot cumulative toxicity study were all obtained from Charles River Laboratories. Authenticated human lung adenocarcinoma cells lines (A549, H358, and H1975) were obtained from American Type Culture Collection (ATCC), maintained according to ATCC protocols, and used within 6 months post-resuscitation. All animal studies were conducted at Lovelace Biomedical under protocols approved by the Lovelace Institutional Animal Care and Use Committee and facilities that are accredited by the Association for Assessment and Accreditation of Laboratory Animal Care (AAALAC) International.

### 2.2. Efficacy of inhaled vs. IV topotecan for orthotopic lung cancer

The efficacy of inhaled vs. IV delivery of topotecan to treat lung cancer was evaluated using our established orthotopic lung cancer model (Belinsky et al., 2011; Reed et al., 2013; Kuehl et al., 2018, 2020). A total of 48 male nude rats were randomized into four treatment groups (Table S1). Group 1 animals (*n* = 6) were kept cancer and treatment naive to serve as age-matched normal control. H358 cells (15 × 10^6^ cells/rat) were instilled via the trachea into the lungs of rats in groups 2–4 and after 3 weeks of tumor growth the rats were treated once-a-week for 4 weeks with vehicle (filtered air), 2 mg/kg topotecan via tail vein, or 1 mg/kg topotecan through inhalation as described (Kuehl et al., 2018). Briefly, the aqueous formulation of topotecan for IV administration was prepared immediately prior to delivery under sterile condition, the injection volume was adjusted based on the body weight of each animal, and injected through the tail vein. The weekly 2 mg/kg IV dose was scaled based on the clinical dose used for small-cell lung cancer patients, 1.5 mg/m^2^/d for five days (Eckardt et al., 2007; von Pawel et al., 2014) or 4–6 mg/m^2^ weekly (Masuda et al., 2010; Allen et al., 2014). The inhalation dose was based on our previous study (Kuehl et al., 2018) and administered using a rodent nose-only inhalation exposure system as described (Reed et al., 2013; Kuehl et al., 2018, 2020). Pulmonary deposited doses were calculated with standard methods using a deposition fraction of 10% (Alexander et al., 2008). All animals were weighed once weekly and sacrificed for moribund conditions or at the end of the study (54 days post-tumor implantation). The lungs from each animal were excised and weighed with tracheas attached. Lung tumor burden for each animal in groups 2–4 was determined by subtracting the average lung weight of the six naïve rats in group 1 from the weight of each tumor-bearing lung. Terminal blood samples collected through cardiac puncture for blood smears and blood counts (complete and differential) were analyzed using the Siemens Advia™ 120 hematology analyzer. Bone marrow, spleen, gastrointestinal tract (GIT), and lung tumors were collected from randomly pre-selected half of the animals in each group and used for histology.

### 2.3. Pilot toxicology evaluation of inhaled topotecan

Hematological toxicity is the major dose limiting toxicity of topotecan in patients and similar toxicity including bone marrow hypocellularity and approximately 10–20% decrease in cell population is seen in Sprague Dawley rats treated with 5 mg/kg topotecan (Davis et al., 2015). Thus, the cumulative toxicity of 1 mg/kg topotecan inhalation once or twice weekly for 4 weeks, which respectively matches or doubles the effective doses used to treat orthotopic lung tumors in rats, was evaluated. A total of 12 Sprague Dawley rats were randomly divided into four groups (three rats/group) and treated for 4 weeks as shown in Table S2. Group 1 animals received vehicle (filtered air) and groups 2–4 received 1 mg/kg of topotecan via inhalation once-a-week (Groups 2 and 3) or twice-a-week (group 4). All animals were observed twice daily for alertness, grooming, feeding, ambulation, breathing, posture, and conditions of the excreta, skin, and fur. Clinical observations including temperature and mucous membrane conditions were evaluated during the weekly body weight measurements. At the end of the study, animals in groups 1, 2, and 4 were sacrificed 24 h after the final (4th) exposure while those in group 3 were sacrificed seven days after the final exposure to assess potential acute toxicities that might resolve or decrease over time. Gross necropsy including examination of external body surfaces, orifices, and the contents of the cranial, thoracic, and abdominal cavities were performed. Blood samples were collected for bioanalytical, hematology, and clinical chemistry via cardiac puncture. Lungs, spleen, brain, and other organs were harvested, weighed, immediately fixed in 10% neutral buffered formalin (NBF), and processed for histology. The frequency and the severity of lesions were evaluated by an experienced veterinary pathologist who was blind to the drug exposures.

### 2.4. Pharmacokinetic analysis of inhaled vs. IV topotecan

The PKs of the therapeutic doses of topotecan used for the treatment of orthotopic lung cancer in rats (2 mg/kg IV and 1 mg/kg inhaled topotecan) have been described (Kuehl et al., 2018). Based on standard inter-species dose scaling approach (Sharma & McNeill, 2009), the 2 mg/kg IV and 1 mg/kg inhalation doses in rat are equivalent to 4 mg/kg and 2 mg/kg in mice, respectively. Considering the significantly better efficacy of inhaled topotecan against orthotopic lung cancer shown in this and our previous (Kuehl et al., 2018) studies and the efficacy of once weekly 10 mg/kg intra-peritoneal topotecan against subcutaneous xenografts in mice (Tessema et al., 2012), 5 mg/kg IV and 1 mg/kg inhaled topotecan doses twice-a-week were selected for efficacy against extrapulmonary tumors in mice. Thus, the current PK study compared 5 mg/kg IV vs. 1 mg/kg inhaled topotecan doses using 60 CD-1^®^ IGS mice (30 mice each for IV and inhalation delivery) as described (Kuehl et al., 2018). Briefly, mice were exposed to 5 mg/kg IV or 1 mg/kg inhaled topotecan and three mice were serially sacrificed from each dose group at 10 time-points over 24 hours (5, 15, and 30 min, 1, 2, 4, 6, 8, 12, and 24 h). At each time-point, systemic blood was collected into K_3_EDTA tubes, the plasma separated, and stored at −80 °C until analysis while lung, liver, and brain tissues were snap frozen on liquid nitrogen. The plasma samples were prepared via a protein precipitation method with 1% formic acid in acetonitrile. The solid tissue samples were homogenized at v/w ratio of one-part tissue to four-parts phosphate-buffered saline (PBS) and underwent the same protein precipitation as the plasma samples prior to analysis using liquid chromatography coupled with tandem mass spectrometry (LC–MS/MS). Topotecan-d6 was used as the internal standard for all samples. Separation was performed with a Waters H-Class UPLC on a C_8_ column (2.1 × 50 mm, 1.7 µm) with a ballistic gradient of 0.1% formic acid in water and 0.1% formic acid in acetonitrile over 3.5 minutes. Quantification was performed in MRM on an ABSciex API 4000 based on matrix based standards between 5 and 5000 ng/mL for all matrices. Linear regression was performed with 1/*x*^2^ weighting for both matrices. Standard bioanalytical matrix based run quality checks (QCs) was also included.

### 2.5. Efficacy of inhaled vs. IV topotecan against extrapulmonary lung tumors

The efficacy of inhaled vs. IV topotecan to treat lung tumors outside of the lungs was evaluated using subcutaneous xenografts in 54 female nude mice randomize into nine groups (six mice/group, Table S3). A549, H358, and H1975 lung cancer cell lines were each mixed one-to-one with Matrigel Basement Membrane Matrix (BD Biosciences, San Jose, CA) and subcutaneously injected on both sides of the dorsal abdomen of mice in groups 1–3 (A549, 2.5 × 10^6^ cells/site), 4–6 (H358, 2.5 × 10^6^ cells/site), and 7–9 (H1975, 1.5 × 10^6^ cells/site). The number of cells/site was lower for H1975 due to its aggressive and fast growth. After 2 weeks of tumor growth, the mice were treated twice-a-week for 6 weeks as follows. Animals in groups 1, 4, and 7 were kept as untreated control for each tumor type and received vehicle (filtered air). Mice in groups 2, 5, and 8 were treated with 5 mg/kg IV topotecan via tail vein and those in groups 3, 6, and 9 were treated with 1 mg/kg topotecan via inhalation. Body weights and tumor size measurements using manual caliper were taken twice-a-week up to the end of the study. Tumor volume was calculated as (*a*×*b*^2^)/2, where *a* and *b* represent the longer and shorter tumor dimensions, respectively, as described (Tessema et al., 2010, 2012). Animals were sacrificed at the end of the study or earlier due to excessive tumor growth or other moribund conditions. A final body weight and tumor size measurement was taken prior to sacrifice, tumors from each flank of the animal were separately removed, weighed, and used for histology. Blood, lung, spleen, and bone marrow were collected from three mice per group for toxicological evaluations.

### 2.6. Statistical analyses

Pharmacokinetic parameters were estimated for plasma, lung, liver and brain using Phoenix WinNonlin version 6.2 software (Certara L.P., Princeton, NJ) using a non-compartmental approach. The area under the concentration vs. time curve (AUC_last_) for each tissue was calculated from time zero to time at the last quantifiable concentration using the linear trapezoidal method with linear/log interpolation. The maximum concentration (*C*_max_) and half-life (*t*_1/2_) of the drug were determined following IV or inhalation delivery. Additional details for the PK analysis method are included in the Supplementary data. Power analysis was used to determine the number of animals per group and our previous studies demonstrated that treatment-related reduction in tumor burden was highly correlated with estimates of tumor volume (Belinsky et al., 2011; Reed et al., 2013; Kuehl et al., 2018, 2020). The two-sample *t*-test and analysis of variance were used to compare the tumor burden between the two treatment groups and each treatment group with the vehicle (control) group. The effects of treatments on the size of subcutaneous xenografts over time were compared using a two-way mixed effect repeated measurement model while the tumor weights were compared using two-sample *t*-test and analysis of variance.

## 3. Results

### 3.1. Inhaled topotecan is more effective in treating orthotopic lung tumors than IV delivery

**Figure 1.**
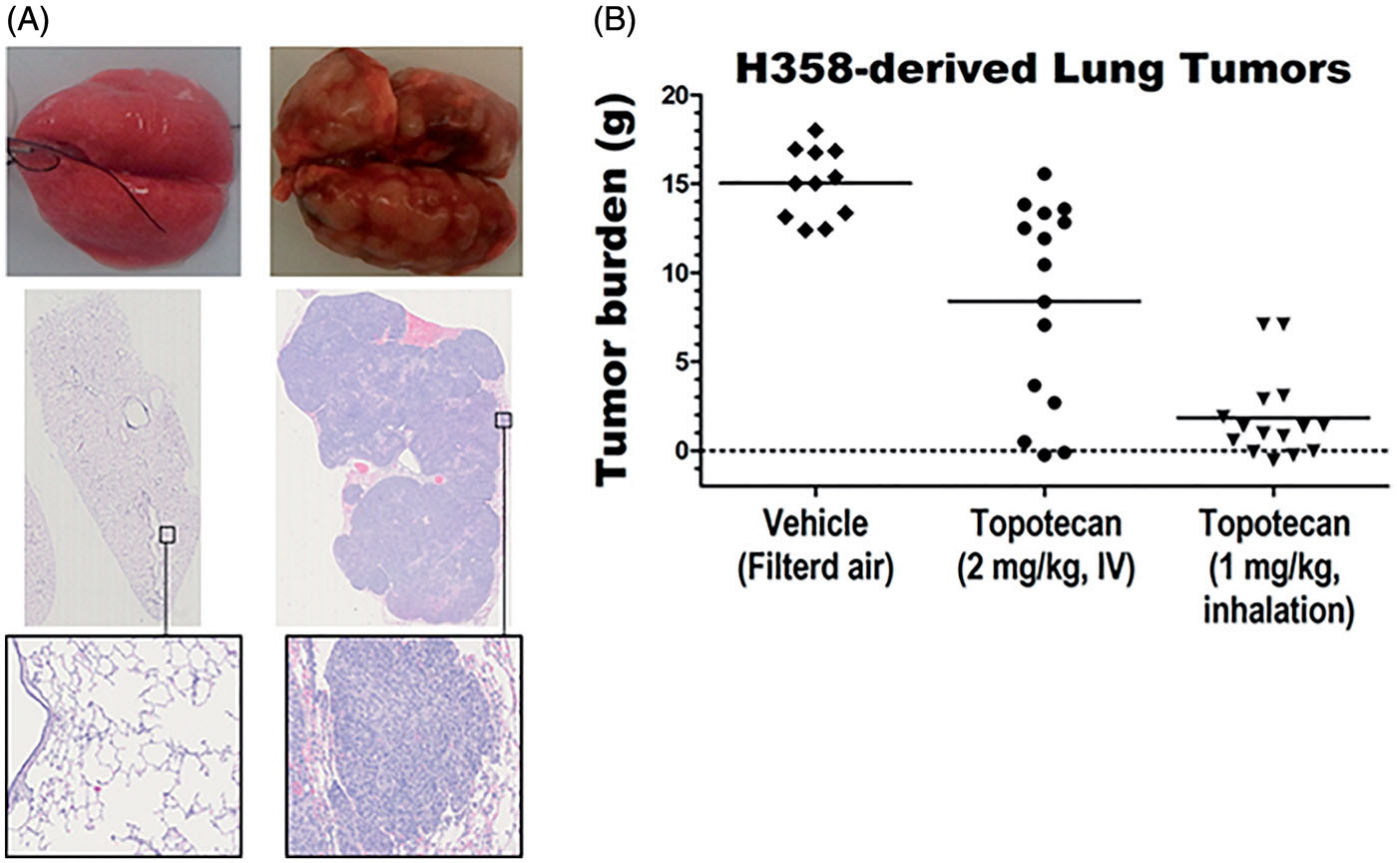
Efficacy of inhaled vs. IV topotecan against orthotopic lung tumors in rats. (A) Gross and histological pictures of lungs from an age-matched normal (left) and H358-derived lung tumors from an untreated control (right) animals. (B) The tumor burden in the three treatment groups.

We have recently demonstrated that inhaled topotecan is significantly more effective against human-derived orthotopic lung cancer in the nude rats compared to the standard IV delivery (Kuehl et al., 2018). Inhalation delivery resulted in significantly better survival of rats with the highly aggressive, EGFR mutant, H1975-derived lung tumors and the tumor burden of the moderately growing, KRAS mutant, A549-derived lung tumors. The current study expanded these findings to a third human NSCLC-derived orthotopic lung cancer model using the EGFR and KRAS wild-type H358 cell line. H358-derived lung tumors grow slightly faster than the A549- but slower than the H1975-derived tumors. The gross and microscopic features of the tumors are shown in Figure 1(A). The average lung tumor burden was significantly lower in both IV- and inhaled-topotecan treated animals compared to the tumor burden of the untreated control (Table 1, Figure 1(B)). Specifically, IV- and inhaled-topotecan respectively reduced the tumor burden by 44% (1.79-fold) and 88% (8.12-fold) than the average 15.02 g tumor burden in the untreated control animals. Most excitingly, the weekly 1 mg/kg inhaled topotecan achieved a significantly better efficacy than the 2 mg/kg IV topotecan (*p*<.00001) by reducing the average tumor burden from 8.40 g to 1.85 g. This demonstrates that inhalation delivery of topotecan dramatically reduced the tumor burden by 78% (4.54-fold) compared to the two times higher dose via the standard IV delivery (Table 1, Figure 1(B)).

**Table 1.** Inhalation delivery of topotecan is more effective in treating H358-derived orthotopic lung tumors compared to two times higher IV dose.

Tumors	Group	Treatments	Lung weight (g)		Tumor burden (g)		*p* Values	
							vs. vehicle	vs. IV-topotecan
			Mean ± SD		Mean ± SD			
None	1	Naïve (*n* = 6)	1.97 ± 0.31				
H358	2	Vehicle (*n* = 12)	16.99 ± 1.97		15.02 ± 1.97			
	3	IV topotecan (2 mg/kg) (*n* = 15)	10.37 ± 5.68		8.40 ± 5.08		5.38E–04*	
	4	Inhaled topotecan (1 mg/kg) (*n* = 15)	3.81 ± 2.38		1.85 ± 2.38		7.93E–14*	5.91E–04*

* Significant differences in tumor-burden between treatment groups.

### 3.2. Topotecan inhalation is well tolerated

The potential for cumulative toxicity of once or twice weekly 1 mg/kg inhaled topotecan that matches or doubles the doses used to effectively treat orthotopic lung cancer were evaluated using Sprague Dawley rats. Body weight of each animal over the treatment period and their lung weights were not significantly different between the treatment groups (Table S4). The minor changes seen in hematology values (Figure S1A,B) largely recovered within seven days and the clinical chemistry results with the exception of reduction in triglycerides were unchanged (Figure S1C–E). Histopathology review revealed a small amount of bronchus-associated lymphoid tissue (BALT) hyperplasia and peribronchiolar/perivascular infiltrates of a few granulocytes. Some lymph nodes in topotecan exposed animals were hyperplastic with increased macrophages and a few sinus granulocytes (not shown). We acknowledge that this toxicology study follows a standard drug development risk-based approach and its goal was limited to ensuring a balance of risk to support an efficacy study. Thus, progress into further drug development needs detailed toxicology focused studies that are outside of the limited scope of the efficacy-oriented data presented in this manuscript.

### 3.3. Pharmacokinetic analysis of topotecan delivered through IV or inhalation

**Figure 2.**
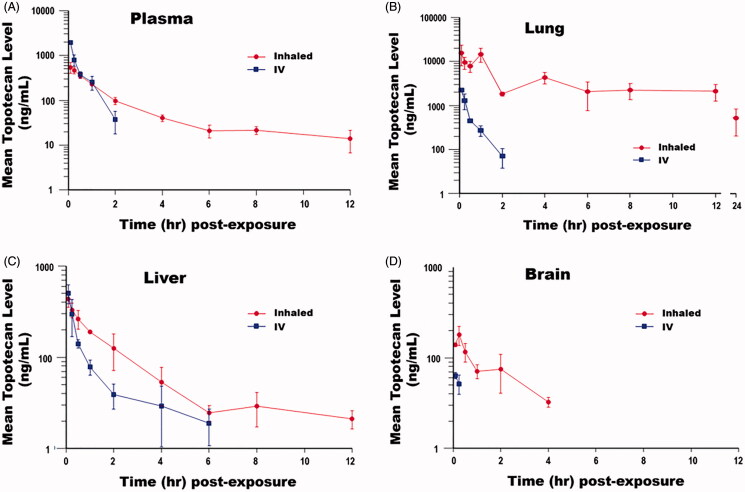
Pharmacokinetic analysis of topotecan following IV or inhalation delivery in mice. The mean levels of topotecan (ng/mL) detected in the (A) plasma, (B) lung, (C) liver, and (D) brain tissues of mice at various time points following 5 mg/kg IV or 1 mg/kg inhalation delivery of the drug.

The PKs of inhaled vs. IV topotecan in rats has been previously evaluated and the results were used to define the therapeutic doses for the treatment of orthotopic lung tumors in the nude rats. Similarly, the PK of 1 mg/kg inhaled vs. 5 mg/kg IV topotecan that were used for the treatment of lung tumors at distant sites outside of the lungs were evaluated using ICR mice. The results showed that topotecan undergoes an apparent bi-phasic elimination profile following inhalation or IV delivery, which is especially evident in plasma and liver (Figure 2(A–D)). The AUC_last_ (h*ng/mL) revealed that inhalation delivery of 1 mg/kg topotecan resulted in 0.94, 55.56, 2.10, and 19.54-fold higher exposure of blood, lung, liver, and brain tissues, respectively, compared to 5 mg/kg topotecan delivered thought IV (Table 2). Similarly, the half-life (*t*_1/2_) of topotecan in all tissues was significantly higher following inhalation delivery than the five times higher IV dose. The *t*_1/2_ of topotecan in the blood, lung, and liver tissue of mice treated via inhalation vs. the IV dose was extended by 13.57-fold (*t*_1/2_=5.89 vs. 0.43 h), 13.33-fold (*t*_1/2_=7.33 vs. 0.56 h), and 1.92-fold (*t*_1/2_=7.34 vs. 3.83 h), respectively. The *t*_1/2_ of topotecan in the brain could not be determined following the IV dose, but lasted 2.45 h after inhalation delivery (Table 2). The PK analysis was done on samples collected at 10 different time-points over 24 h period following single IV or inhaled exposure. However, the topotecan level in the IV group dropped below the detection limit of 5 ng/mL LLOQ at the later time-points due to rapid clearance from the circulation and may explain why the AUC value is lower than the *C*_max_ for this group. Although this highlights one of the major disadvantages of IV topotecan, it has also limited the completeness of the terminal PK analysis of IV topotecan.

**Table 2.** Pharmacokinetics profile of topotecan following inhalation or IV delivery.

Tissue analyzed	Route of delivery	Dose (mg/kg)	*C*_max_±SE (ng/mL)	AUC_last_±SE (h*ng/mL)	*t*_1/2_ (h)
Plasma	IV	5.0	1960 ± 223	888 ± 87.6	0.434
	Inhalation	1.0	542 ± 144	831 ± 50.0	5.89
Lung	IV	5.0	2240 ± 179	1080 ± 119	0.556
	Inhalation	1.0	15,600 ± 7680	60,000 ± 9350***	7.33
Liver	IV	5.0	5040 ± 1170	4000 ± 539	3.83
	Inhalation	1.0	4360 ± 821	8380 ± 1060*	7.34
Brain	IV	5.0	63.5 ± 4.86	15.2 ± 1.19	NC
	Inhalation	1.0	180 ± 821	297 ± 54.3*	2.45

NC: not calculated because values quickly dropped below detection limit.

*p< .05, ***p< .001 using linear model to compare the differences in each tissue by the route of delivery.

### 3.4. Inhaled topotecan suppresses the growth of lung tumors at distant sites

**Figure 3.**
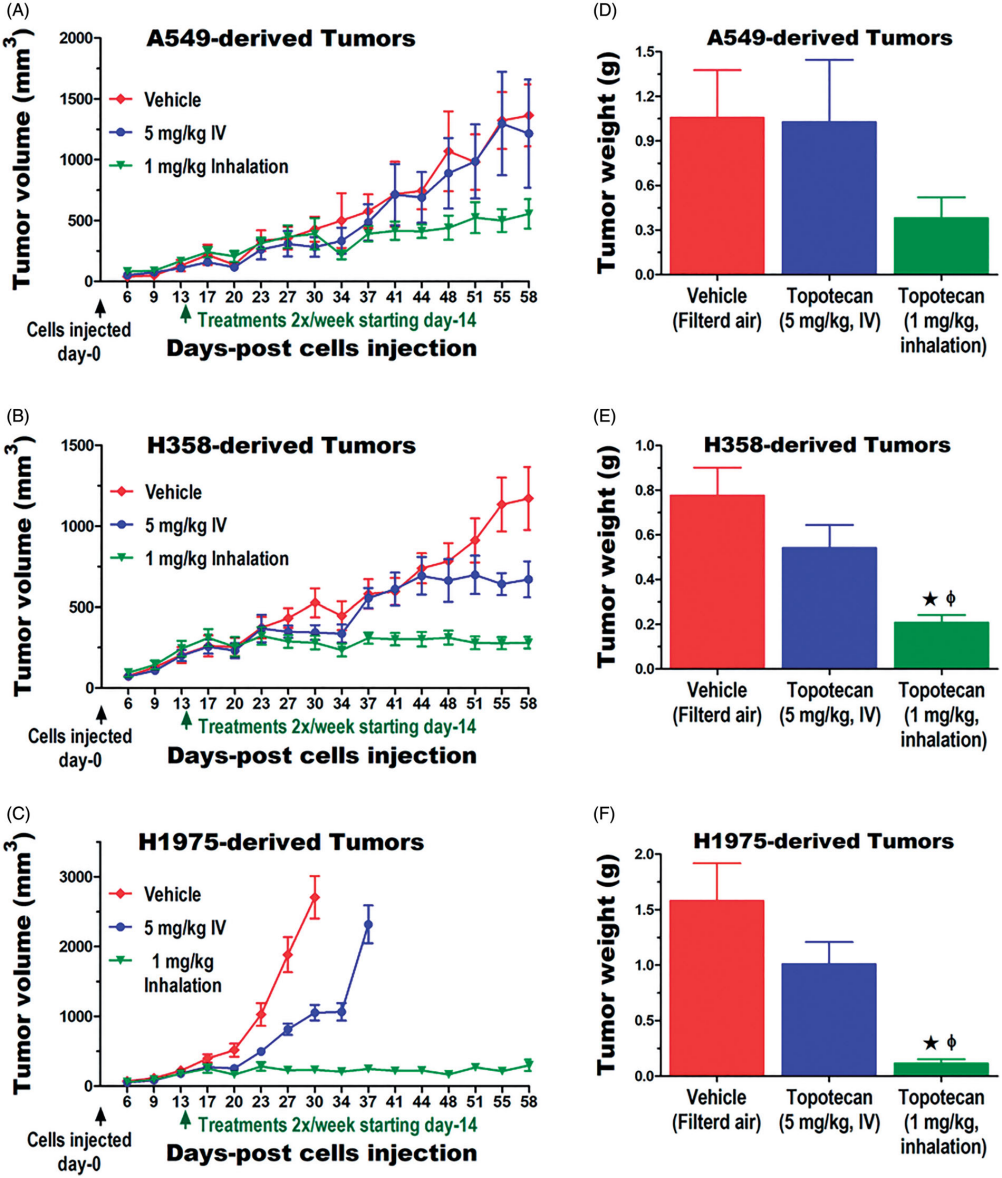
Inhalation delivery of topotecan leads to superior efficacy against lung tumors at distant sites than IV delivery. The growth of tumors derived from (A) the KRAS mutant A549, (B) the KRAS and EGFR wildtype H358, and (C) the EGFR mutant H1975 human NSCLC cell lines revealed a superior efficacy of 1 mg/kg inhaled topotecan compared to 5 mg/kg IV topotecan. (D, E) The average weights of these tumors obtained at the end of the study further confirmed the tumor measurement data. Significant differences (*p*<.05) from vehicle control and IV topotecan treated groups are shown as * and ϕ, respectively.

The efficacy of inhaled topotecan in suppressing the growth of tumors outside of the lungs was compared with the standard IV delivery using a well-established subcutaneous xenograft model in the nude mouse. We acknowledge that the growth of lung tumors as subcutaneous xenografts does not truly represent metastatic cancer. However, in the absence of a reliable lung cancer metastasis model, it provides proof-of-concept whether inhaled topotecan deliver optimum doses that can effectively suppress tumor growth at distant sites. Thus, the three human NSCLC cell lines (A549, H358, and H1975) used in the orthotopic model in rats were used to generate subcutaneous xenografts in nude mice. Mice treated with 1 mg/kg inhaled topotecan showed the slowest growth while those in the vehicle control group showed the fastest tumor growth of all three tumor types (Figure 3(A–C)). In contrast, 5 mg/kg IV topotecan moderately suppressed the growth of H1975 and H358-derived tumors but had no effect on the growth of A549-derived xenografts. The tumor weight data at sacrifice confirmed these measurements. Specifically, the average tumor weight for each of the tumor types was the lowest in mice treated via inhalation and the highest among the animals in the control groups (Figure 3(D–F)). Most exciting, the weights of A549, H358, and H1975-derived tumors in mice treated with 1 mg/kg inhaled topotecan were 2.7, 2.6, and 8.8-fold lower compared to those treated with the 5 mg/kg IV dose (Figure 3(D–F)). The gross pictures of some tumors at the end of the study taken immediately before and after they were harvested are shown in Figure S2A–F.

## 4. Discussion

This study demonstrates the superior efficacy of inhaled topotecan against lung cancer within and outside of the lungs compared to IV delivery, the current standard and FDA-approved use of this drug. These findings reveal the unique advantages of inhaled topotecan, support expanding its clinical use, and could lead to a paradigm shift in the use of inhalation therapy for the treatment of lung cancer. We have recently shown that inhalation delivery of topotecan is more effective in suppressing orthotopic growth of EGFR and KRAS mutant lung tumors compared to a two times higher IV dose (Kuehl et al., 2018). In this study, those findings were reproduced using a 3rd lung cancer model that is wild-type to both EGFR and KRAS oncogenes, thereby confirming the wider efficacy of inhaled topotecan against tumors that vary based on the two most common cancer-driver oncogenes in lung cancer. Moreover, inhalation delivery of topotecan is also more effective in suppressing extrapulmonary growth of all three lung tumor types at one-fifth of the IV dose. This enhanced potency stemmed from a superior PK profile of inhaled topotecan that resulted in better exposure and longer half-life of the drug in the systemic circulation as well as lung, liver, and brain tissues at lower doses than IV infusion. Taken together, the superior efficacy and PKs of inhaled topotecan offer the potential to reduce toxicity and improve efficacy for the treatment of local and metastatic lung cancer compared to the current protocol.

The importance of inhalation delivery of drugs to bypass the inactivating effects of digestive and hepatic enzymes and treat diseases primarily affecting the respiratory system such as asthma and COPD is well known. Various inhalable drugs have already been approved for these diseases and many others are currently in clinical trials (Mann & Meyer, 2018; Donohue et al., 2019; Rabe et al., 2020; Voelker, 2020). Inhalation delivery achieves higher drug concentrations in the lungs with considerably lower systemic maximum concentration (*C*_max_), thus significantly minimizing potential for systemic toxicity. This also improves efficacy of some drugs at least locally by allowing delivery of higher concentrations to the lungs that would otherwise be impossible via systemic delivery due to dose limiting toxicities. As shown in our toxicological assessment, the dose limiting hematopoietic toxicities of topotecan that prevent the use of its most effective dose and wider clinical application could be mitigated through inhalation delivery. Our laboratory is currently trying to exploit these advantages of inhalation delivery for the treatment of primary and local metastasis of lung cancer while others investigate its potential use for cancers that are commonly metastasize into the lungs. Chemotherapeutic agents evaluated using inhalation delivery in clinical and/or pre-clinical settings and demonstrating some degree of efficacy and tolerability include cisplatin, doxorubicin, 9-nitro-campthotecin, and gemcitabine (Koshkina et al., 2000; Verschraegen et al., 2004; Koshkina & Kleinerman, 2005; Otterson et al., 2007; Wittgen et al., 2007).

In contrast, the potential use of inhalation delivery for the treatment of tumors outside of the lungs is not well studied. This may be, in part, due to the poor systemic bioavailability of some active pharmaceutical ingredients (APIs) or the drug product utilized. As we have recently demonstrated for the epigenetic drug 5-azacytidine (Kuehl et al., 2020), the poor systemic distribution of some inhaled drugs may also be related to the aqueous nebulized formulation rather than the route of administration. Our findings in the previous study where inhalation delivery of 5-azacytidine dry powder significantly increased the systemic distribution compared to inhalation of the aqueous nebulized formulation clearly supporting this premise. In this study, we showed that the terminal half-life (*t*_1/2_) of topotecan following IV delivery is much shorter in the various tissues evaluated compared to inhalation delivery. This suggests a flip-flop model where the systemic clearance following inhalation delivery is partly compensated by absorption of topotecan from the lungs. This likely contributed to the increased terminal *t*_1/2_ half-life as well as the extended exposure both locally and systemically. A similar observation using inhalation delivery of another anticancer drug paclitaxel has been recently reported (Verco et al., 2019). Overall, the mechanism(s) responsible for the increased systemic PK profile and the extrapulmonary anticancer efficacy of inhaled topotecan over IV delivery are not yet clearly defined. However, the following general or topotecan-specific advantages of dry powder formulation and inhalation delivery likely played a role. Among these, the relative improved stability of many drugs in dry powder compared to aqueous form, the potential for dissolution rate limited release of inhaled drugs into the systemic circulation that slows the rate of excretion and detoxification by hepatic enzymes are potential mechanisms that may apply to many drugs. The superior systemic profile and local anticancer efficacy of inhaled 5-azacytidine dry powder supports this premise (Kuehl et al., 2020).

In addition, the dry powder formulation and inhalation delivery directly into the lungs also provide some unique advantages to topotecan. The anticancer activity of the CPT family of topoisomerase-I inhibitors including the two FDA approved drugs topotecan and irinotecan (CPT-11) rely on the lactone form of each drug that is critical for binding to topoisomerase-I (Venditto & Simanek, 2010). Rapid hydrolysis of the lactone form in solution to a carboxylate form leads to complete loss of activity. The equilibrium between the active lactone and the inactive carboxylate forms is influenced by the pH of the solution and affinity of the carboxylate for human serum albumin. Under physiological pH at 37 °C, the carboxylate form is predominant and equilibrium is reached quickly, for example within 90 min following IV injection of either 1 mg/kg lactone or carboxylate in rats (Gabr et al., 1997; Venditto & Simanek, 2010). Thus, when these drugs are delivered via IV, only 35% of topotecan and 37% of CPT-11 are active in human blood (Venditto & Simanek, 2010). Unlike CPT-11, which is a prodrug and needs activation by hepatic enzymes, topotecan is an active drug uniquely suited for dry powder formulation and inhalation delivery. Taken together, these general and unique advantages of the dry powder formulation and inhalation delivery likely contributed to the better systemic PK profile and efficacy of inhaled topotecan.

We and others have shown that the sensitivity of lung and other cancers to topotecan and its parent compound CPT is associated with expression of *Interferon-stimulated gene 15* (*ISG15*) (Desai et al., 2006, 2008; Tessema et al., 2012). Tumors from high *ISG15* expressing lung cancer cells such as H1975 and H358 are more sensitive to topotecan and CPT while those expressing low *ISG15* such as A549 are less sensitive to these drugs. In agreement with this, our current study also identified similar differences in the efficacy of inhaled and IV topotecan against tumors with low or high *ISG15* expression. Subcutaneous xenografts derived from the two high *ISG15* expressing lung cancer cell lines (H1975 and H358) showed higher sensitivity to inhaled and to a lesser extent IV topotecan compared to the moderate or no response seen in the low *ISG15* expressing A549-derived tumors. In contrast, inhalation delivery of topotecan dramatically reduced the orthotopic growth of all three lung cancer types regardless of high or low *ISG15* expression. This may indicate that the high concentration of topotecan deposited in the lungs following inhalation delivery could overcome even the less sensitive A549-derived tumors (Tessema et al., 2012; Kuehl et al., 2018). In fact, we have shown that inhalation delivery of two times lower dose of topotecan resulted in approximately 30-fold higher concentration of the drug within the lungs compared to IV delivery (Kuehl et al., 2018). This suggests that lung cancer patients who have tumors that are normally insensitive to topotecan and potentially other chemotherapeutics may similarly benefit from the increased concentration of the drug(s) achieved through inhalation delivery. Moreover, the broad spectrum of tumors responding to inhaled topotecan may offer additional therapies following progression of EGFR or KRAS mutant cancers.

## Supplementary Material

Supplemental Material

## References

[CIT0001] Alexander DJ, Collins CJ, Coombs DW, et al. (2008). Association of Inhalation Toxicologists (AIT) Working Party Recommendation for standard delivered dose calculation and expression in non-clinical aerosol inhalation toxicology studies with pharmaceuticals. Inhal Toxicol 20:1179–89. 18802802 10.1080/08958370802207318

[CIT0002] Allen JW, Moon J, Redman M, et al. (2014). Southwest Oncology Group S0802: a randomized, phase II trial of weekly topotecan with and without ziv-aflibercept in patients with platinum-treated small-cell lung cancer. J Clin Oncol 32:2463–70. 25002722 10.1200/JCO.2013.51.4109 PMC4121504

[CIT0003] Belinsky SA, Grimes MJ, Picchi MA, et al. (2011). Combination therapy with vidaza and entinostat suppresses tumor growth and reprograms the epigenome in an orthotopic lung cancer model. Cancer Res 71:454–62. 21224363 10.1158/0008-5472.CAN-10-3184 PMC3075424

[CIT0004] Brahmer JR, Tykodi SS, Chow LQ, et al. (2012). Safety and activity of anti-PD-L1 antibody in patients with advanced cancer. N Engl J Med 366:2455–65. 22658128 10.1056/NEJMoa1200694 PMC3563263

[CIT0005] Bray F, Ferlay J, Soerjomataram I, et al. (2018). Global cancer statistics 2018: GLOBOCAN estimates of incidence and mortality worldwide for 36 cancers in 185 countries. CA Cancer J Clin 68:394–424. 30207593 10.3322/caac.21492

[CIT0006] Camidge DR, Pao W, Sequist LV. (2014). Acquired resistance to TKIs in solid tumours: learning from lung cancer. Nat Rev Clin Oncol 11:473–81. 24981256 10.1038/nrclinonc.2014.104

[CIT0007] Carbone DP, Reck M, Paz-Ares L, et al. (2017). First-line nivolumab in stage IV or recurrent non-small-cell lung cancer. N Engl J Med 376:2415–26. 28636851 10.1056/NEJMoa1613493 PMC6487310

[CIT0008] Davis M, Li J, Knight E, et al. (2015). Toxicogenomics profiling of bone marrow from rats treated with topotecan in combination with oxaliplatin: a mechanistic strategy to inform combination toxicity. Front Genet 6:14. 25729387 10.3389/fgene.2015.00014 PMC4325931

[CIT0009] Desai SD, Haas AL, Wood LM, et al. (2006). Elevated expression of ISG15 in tumor cells interferes with the ubiquitin/26S proteasome pathway. Cancer Res 66:921–8. 16424026 10.1158/0008-5472.CAN-05-1123

[CIT0010] Desai SD, Wood LM, Tsai YC, et al. (2008). ISG15 as a novel tumor biomarker for drug sensitivity. Mol Cancer Ther 7:1430–9. 18566215 10.1158/1535-7163.MCT-07-2345 PMC2561335

[CIT0011] Donohue JF, Mahler DA, Sethi S. (2019). Revefenacin: a once-daily, long-acting bronchodilator for nebulized treatment of COPD. Int J Chron Obstruct Pulmon Dis 14:2947–58. 31908443 10.2147/COPD.S157654 PMC6927563

[CIT0012] Eckardt JR, von Pawel J, Pujol JL, et al. (2007). Phase III study of oral compared with intravenous topotecan as second-line therapy in small-cell lung cancer. J Clin Oncol 25:2086–92. 17513814 10.1200/JCO.2006.08.3998

[CIT0013] Gabr A, Kuin A, Aalders M, et al. (1997). Cellular pharmacokinetics and cytotoxicity of camptothecin and topotecan at normal and acidic pH. Cancer Res 57:4811–6. 9354443

[CIT0014] Gandhi L, Rodriguez-Abreu D, Gadgeel S, et al. (2018). Pembrolizumab plus chemotherapy in metastatic non-small-cell lung cancer. N Engl J Med 378:2078–92. 29658856 10.1056/NEJMoa1801005

[CIT0015] Jones S, Thompson D, Barton J, et al. (2008). A randomized phase II trial of oral topotecan versus docetaxel in the second-line treatment of non-small-cell lung cancer. Clin Lung Cancer 9:154–9. 18621625 10.3816/CLC.2008.n.023

[CIT0016] Kindler HL, Kris MG, Smith IE, et al. (1998). Phase II trial of topotecan administered as a 21-day continuous infusion in previously untreated patients with stage IIIB and IV non-small-cell lung cancer. Am J Clin Oncol 21:438–41. 9781595 10.1097/00000421-199810000-00003

[CIT0017] Koshkina NV, Kleinerman ES. (2005). Aerosol gemcitabine inhibits the growth of primary osteosarcoma and osteosarcoma lung metastases. Int J Cancer 116:458–63. 15800950 10.1002/ijc.21011

[CIT0018] Koshkina NV, Kleinerman ES, Waidrep C, et al. (2000). 9-Nitrocamptothecin liposome aerosol treatment of melanoma and osteosarcoma lung metastases in mice. Clin Cancer Res 6:2876–80. 10914737

[CIT0019] Kuehl PJ, Grimes MJ, Dubose D, et al. (2018). Inhalation delivery of topotecan is superior to intravenous exposure for suppressing lung cancer in a preclinical model. Drug Deliv 25:1127–36. 29779406 10.1080/10717544.2018.1469688 PMC6058531

[CIT0020] Kuehl PJ, Tellez CS, Grimes MJ, et al. (2020). 5-Azacytidine inhaled dry powder formulation profoundly improves pharmacokinetics and efficacy for lung cancer therapy through genome reprogramming. Br J Cancer 122:1194–204. 32103148 10.1038/s41416-020-0765-2 PMC7156464

[CIT0021] Lynch TJ, Jr., Kalish L, Strauss G, et al. (1994). Phase II study of topotecan in metastatic non-small-cell lung cancer. J Clin Oncol 12:347–52. 8113842 10.1200/JCO.1994.12.2.347

[CIT0022] Mann M, Meyer RJ. (2018). Drug development for asthma and COPD: a regulatory perspective. Respir Care 63:797–817. 29794212 10.4187/respcare.06009

[CIT0023] Martin P, Leighl NB, Tsao MS, et al. (2013). KRAS mutations as prognostic and predictive markers in non-small cell lung cancer. J Thorac Oncol 8:530–42. 23524403 10.1097/JTO.0b013e318283d958

[CIT0024] Masuda N, Matsui K, Negoro S, et al. (2010). Phase I and pharmacologic study of weekly bolus topotecan for advanced non-small-cell lung cancer. Clin Lung Cancer 11:271–9. 20630830 10.3816/CLC.2010.n.035

[CIT0025] Meng X, Liu Y, Zhang J, et al. (2017). PD-1/PD-L1 checkpoint blockades in non-small cell lung cancer: new development and challenges. Cancer Lett 405:29–37. 28688973 10.1016/j.canlet.2017.06.033

[CIT0026] O'Brien ME, Ciuleanu TE, Tsekov H, et al. (2006). Phase III trial comparing supportive care alone with supportive care with oral topotecan in patients with relapsed small-cell lung cancer. J Clin Oncol 24:5441–7. 17135646 10.1200/JCO.2006.06.5821

[CIT0027] O'Brien M, Eckardt J, Ramlau R. (2007). Recent advances with topotecan in the treatment of lung cancer. Oncologist 12:1194–204. 17962613 10.1634/theoncologist.12-10-1194

[CIT0028] Otterson GA, Villalona-Calero MA, Sharma S, et al. (2007). Phase I study of inhaled doxorubicin for patients with metastatic tumors to the lungs. Clin Cancer Res 13:1246–52. 17317836 10.1158/1078-0432.CCR-06-1096

[CIT0029] Paez JG, Janne PA, Lee JC, et al. (2004). EGFR mutations in lung cancer: correlation with clinical response to gefitinib therapy. Science 304:1497–500. 15118125 10.1126/science.1099314

[CIT0030] Pao W, Girard N. (2011). New driver mutations in non-small-cell lung cancer. Lancet Oncol 12:175–80. 21277552 10.1016/S1470-2045(10)70087-5

[CIT0031] Perez-Soler R, Fossella FV, Glisson BS, et al. (1996). Phase II study of topotecan in patients with advanced non-small-cell lung cancer previously untreated with chemotherapy. J Clin Oncol 14:503–13. 8636764 10.1200/JCO.1996.14.2.503

[CIT0032] Rabe KF, Martinez FJ, Ferguson GT, et al. (2020). Triple inhaled therapy at two glucocorticoid doses in moderate-to-very-severe COPD. N Engl J Med 383:35–48. 32579807 10.1056/NEJMoa1916046

[CIT0033] Ramlau R, Gervais R, Krzakowski M, et al. (2006). Phase III study comparing oral topotecan to intravenous docetaxel in patients with pretreated advanced non-small-cell lung cancer. J Clin Oncol 24:2800–7. 16682727 10.1200/JCO.2005.03.6491

[CIT0034] Reed MD, Tellez CS, Grimes MJ, et al. (2013). Aerosolised 5-azacytidine suppresses tumour growth and reprogrammes the epigenome in an orthotopic lung cancer model. Br J Cancer 109:1775–81. 24045660 10.1038/bjc.2013.575 PMC3790193

[CIT0035] Ribas A, Wolchok JD. (2018). Cancer immunotherapy using checkpoint blockade. Science 359:1350–5. 29567705 10.1126/science.aar4060 PMC7391259

[CIT0036] Sharma V, McNeill JH. (2009). To scale or not to scale: the principles of dose extrapolation. Br J Pharmacol 157:907–21. 19508398 10.1111/j.1476-5381.2009.00267.x PMC2737649

[CIT0037] Siegel RL, Miller KD, Jemal A. (2020). Cancer statistics, 2020. CA A Cancer J Clin 70:7–30. 10.3322/caac.21590 31912902

[CIT0038] Socinski MA, Jotte RM, Cappuzzo F, et al. (2018). Atezolizumab for first-line treatment of metastatic nonsquamous NSCLC. N Engl J Med 378:2288–301. 29863955 10.1056/NEJMoa1716948

[CIT0039] Soda M, Choi YL, Enomoto M, et al. (2007). Identification of the transforming EML4-ALK fusion gene in non-small-cell lung cancer. Nature 448:561–6. 17625570 10.1038/nature05945

[CIT0040] Tessema M, Klinge DM, Yingling CM, et al. (2010). Re-expression of CXCL14, a common target for epigenetic silencing in lung cancer, induces tumor necrosis. Oncogene 29:5159–70. 20562917 10.1038/onc.2010.255 PMC2940978

[CIT0041] Tessema M, Yingling CM, Thomas CL, et al. (2012). SULF2 methylation is prognostic for lung cancer survival and increases sensitivity to topoisomerase-I inhibitors via induction of ISG15. Oncogene 31:4107–16. 22158045 10.1038/onc.2011.577 PMC3307938

[CIT0042] Tsao MS, Sakurada A, Cutz JC, et al. (2005). Erlotinib in lung cancer – molecular and clinical predictors of outcome. N Engl J Med 353:133–44. 16014883 10.1056/NEJMoa050736

[CIT0043] Venditto VJ, Simanek EE. (2010). Cancer therapies utilizing the camptothecins: a review of the in vivo literature. Mol Pharm 7:307–49. 20108971 10.1021/mp900243b PMC3733266

[CIT0044] Verco J, Johnston W, Baltezor M, et al. (2019). Pharmacokinetic profile of inhaled submicron particle paclitaxel (NanoPac^®^) in a rodent model. J Aerosol Med Pulm Drug Deliv 32:99–109. 30359162 10.1089/jamp.2018.1467 PMC6477588

[CIT0045] Verschraegen CF, Gilbert BE, Loyer E, et al. (2004). Clinical evaluation of the delivery and safety of aerosolized liposomal 9-nitro-20(s)-camptothecin in patients with advanced pulmonary malignancies. Clin Cancer Res 10:2319–26. 15073107 10.1158/1078-0432.ccr-0929-3

[CIT0046] Voelker R. (2020). Generic albuterol inhaler approved. JAMA 323:1887. 10.1001/jama.2020.7282 32427293

[CIT0047] von Pawel J, Jotte R, Spigel DR, et al. (2014). Randomized phase III trial of amrubicin versus topotecan as second-line treatment for patients with small-cell lung cancer. J Clin Oncol 32:4012–9. 25385727 10.1200/JCO.2013.54.5392

[CIT0048] Wang C, Qiao W, Jiang Y, et al. (2020). The landscape of immune checkpoint inhibitor plus chemotherapy versus immunotherapy for advanced non-small-cell lung cancer: a systematic review and meta-analysis. J Cell Physiol 235:4913–27. 31693178 10.1002/jcp.29371 PMC7028135

[CIT0049] Weitz JJ, Marschke RF, Jr., Sloan JA, et al. (2000). A randomized phase II trial of two schedules of topotecan for the treatment of advanced stage non-small cell lung cancer. Lung Cancer 28:157–62. 10717333 10.1016/s0169-5002(99)00128-2

[CIT0050] West H, McCleod M, Hussein M, et al. (2019). Atezolizumab in combination with carboplatin plus nab-paclitaxel chemotherapy compared with chemotherapy alone as first-line treatment for metastatic non-squamous non-small-cell lung cancer (IMpower130): a multicentre, randomised, open-label, phase 3 trial. Lancet Oncol 20:924–37. 31122901 10.1016/S1470-2045(19)30167-6

[CIT0051] Wittgen BP, Kunst PW, van der Born K, et al. (2007). Phase I study of aerosolized SLIT cisplatin in the treatment of patients with carcinoma of the lung. Clin Cancer Res 13:2414–21. 17438100 10.1158/1078-0432.CCR-06-1480

