# Supplementary material for: Inhalation delivery dramatically improves the efficacy of topotecan for the treatment of local and distant lung cancer
Source: Drug Deliv. 2021 Apr 16;28(1):767–75. doi: 10.1080/10717544.2021.1912209 (PMC8079036; doi:10.1080/10717544.2021.1912209)
Supplement: Supplemental Material [file IDRD_A_1912209_SM7542.zip › Supplementary Data.docx]

**Supplementary Data**

**Supplementary Tables**

**Table S1: Experimental Design for the Treatment of Orthotopic Lung Tumors in Rats**

| **Group** | **Lung cancer Cell line** | **Number of rats** | **Treatments** ** | | |
| --- | --- | --- | --- | --- | --- |
|  |  |  | **Vehicle** | **2 mg/kg IV** | **1 mg/kg inhalation** |
| 1 | None * | 6 | - | - | - |
| 2 | H358 | 12 | + | - | - |
| 3 |  | 15 | - | + | - |
| 4 |  | 15 | - | - | + |

* Animals in Group 1 serve as age matched cancer-free and treatment-free control.

** The ‘+’ and ‘-‘ signs indicate the treatments given or not given, respectively.

**Table S2: Experimental Design for the Pilot Cumulative Toxicity study in Rats**

| **Group** | **Number of Rats** | **Topotecan Inhalation** | **Necropsy** |
| --- | --- | --- | --- |
| 1 | 3 | NA | 24 h after the last dose |
| 2 | 3 | 1 mg/kg, 1X/week | 24 h after the last dose |
| 3 | 3 | 1 mg/kg, 1X/week | 7 days after the last dose |
| 4 | 3 | 1 mg/kg, 2X/week | 24 h after the last dose |

**Table S3: Experimental Design for the Treatment of NSCLC Subcutaneous Xenografts in Mice**

| **Group** | **NSCLC Cell line** | **Number of mice** | **Treatments** | |
| --- | --- | --- | --- | --- |
|  |  |  | **Route** | **Dose** |
| 1 | A549 | 6 | None | None |
| 2 |  | 6 | TPT IV | 5 mg/kg, 2X/week |
| 3 |  | 6 | TPT Inhalation | 1 mg/kg, 2X/week |
| 4 | H358 | 6 | None | None |
| 5 |  | 6 | TPT IV | 5 mg/kg, 2X/week |
| 6 |  | 6 | TPT Inhalation | 1 mg/kg, 2X/week |
| 7 | H1975 | 6 | None | None |
| 8 |  | 6 | TPT IV | 5 mg/kg, 2X/week |
| 9 |  | 6 | TPT Inhalation | 1 mg/kg, 2X/week |

**Table S4: The therapeutic doses of inhaled topotecan cause no local or systemic toxicity**

| **Animals and treatments** | | | **Necropsy (post-final dose)** | **Body weight gains (g, mean ± SD) ^‡^** | **Lung weight (g)**  **(mean ± SD)** |
| --- | --- | --- | --- | --- | --- |
| **Groups** | **Treatments (4 weeks) ^†^** | |  |  |  |
| 1 (n = 3) | Filtered air | 1 x weekly | 24 hours | 151.87 ± 9.71 | 1.76 ± 0.29 |
| 2 (n = 3) | Topotecan | 1 x weekly | 24 hours | 115.17 ± 21.41 | 1.79 ± 0.15 |
| 3 (n = 3) | Topotecan | 1 x weekly | 7 days | 147.51 ± 19.80 | 2.00 ± 0.13 |
| 4 (n = 3) | Topotecan | 2 x weekly | 24 hours | 93.10 ± 28.59 | 1.75 ± 0.25 |

**^†^** All animals were treated with 1 mg/kg body weight topotecan through nose-only inhalation.

**^‡^** Body weight gains of the animals over the over the treatment period.

**Supplementary Figure legends**

**Figure S1: Hematology and clinical chemistry results show that the therapeutic doses of topotecan inhalation are well tolerated.** Hematology and clinical chemistry evaluation was conducted on blood samples from rats exposed to the therapeutic doses of 1 mg/kg inhaled topotecan once or twice-a-week for 4 weeks. The results for some of the measurements including **(A)** total and differential white blood cell counts, **(B)** hepatic panel, **(C)** basic metabolic panel, **(D)** platelet count, and **(E)** lipid panel are shown. Blood samples were taken 24 hours after the final (4^th^) exposure of Vehicle (Group-1), 1 mg/kg topotecan once weekly (Group-2), twice weekly (Group-4), or 7 days after the final (4^th^) exposure of 1 mg/kg topotecan (Group-3).

**Figure S2: Gross pictures of A549- and H358-derived tumors from mice in the three treatment groups before and after collection.** The gross pictures of **(A − C)** A549- and **(D − F)** H358-derived tumors were taken before (top) or after (bottom) collection from (**A** and **D**) Vehicle, (**B** and **E**) 5 mg/kg IV topotecan, or (**C** and **F**) 1 mg/kg inhaled topotecan treated mice are shown**.**

**Supplementary Methods**

**Pharmacokinetic (PK) analysis**

Pharmacokinetic parameters were estimated for plasma, lung, brain and liver using Phoenix WinNonlin version 6.2 software (Certara L.P.). A non-compartmental approach consistent with the route of administration (IV Bolus for intravenous dose, extravascular model for inhaled dose) and sparse sampling were used since each subject was sampled at only one time-point. All parameters were generated from composite topotecan concentrations. Concentrations that were below the quantification limit (BQL) were treated as missing for the analysis. For group/time-points where only one of three samples was able to be quantified (and the other two samples for that group/timepoint were BQL), the quantified sample was excluded from analysis.

The area under the concentration vs time curve (AUClast) for each tissue, from time zero to the time at which the last quantifiable concentration was observed, was calculated using the linear trapezoidal method with linear/log interpolation and presented with standard error. Additional parameters generated by Phoenix WinNonlin and presented here include maximum observed concentration (Cmax) with standard error, and dose-normalized AUClast and Cmax. Additional parameters were automatically generated by Phoenix and are maintained in the data.

After data visualization, biphasic systemic clearance was apparent, especially in the liver and plasma tissue. The latter, or beta, terminal elimination phase of each concentration versus time curve was identified using at least the final three observed concentration values in Phoenix WinNonlin. The initial clearance, or alpha, phase was identified with the final time-point equal to the initial time-point of the beta phase, and at least the two previous concentrations included. The slope of both terminal elimination phases was determined using log regression with uniform weighting. Alpha and beta terminal elimination half-life were reported if the reporting criteria were met: the coefficient of determination (R2) was greater than or equal to 0.6 and the extrapolation of the AUC to infinity was less than or equal to 30% of the total area.

Linear mixed modeling in Phoenix WinNonlin was used to test the effect of the delivery method (Inhaled vs IV) separately in each tissue. Concentration from the initial clearance phase (time-points from 0 to 6 hours) was used, and concentrations were log-normalized. Terms for time, tissue, delivery method, and the interaction between tissue and delivery method were included to predict concentration in the model.
