# Supplementary figures and images for: Inhalation delivery dramatically improves the efficacy of topotecan for the treatment of local and distant lung cancer
Source: Drug Deliv. 2021 Apr 16;28(1):767–75. doi: 10.1080/10717544.2021.1912209 (PMC8079036; doi:10.1080/10717544.2021.1912209)

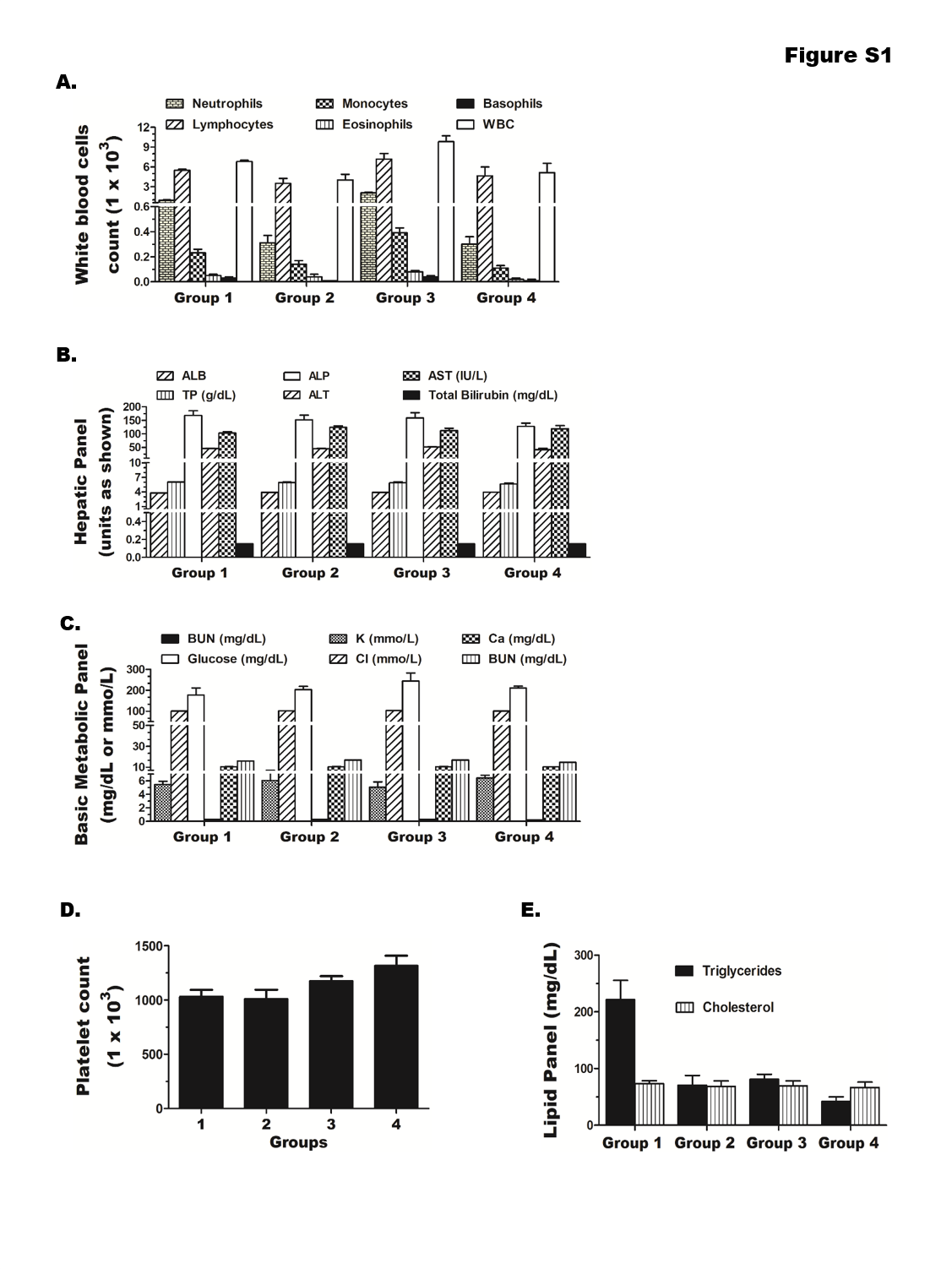

Supplement: Supplemental Material [file IDRD_A_1912209_SM7542.zip › Figure S1.tif]

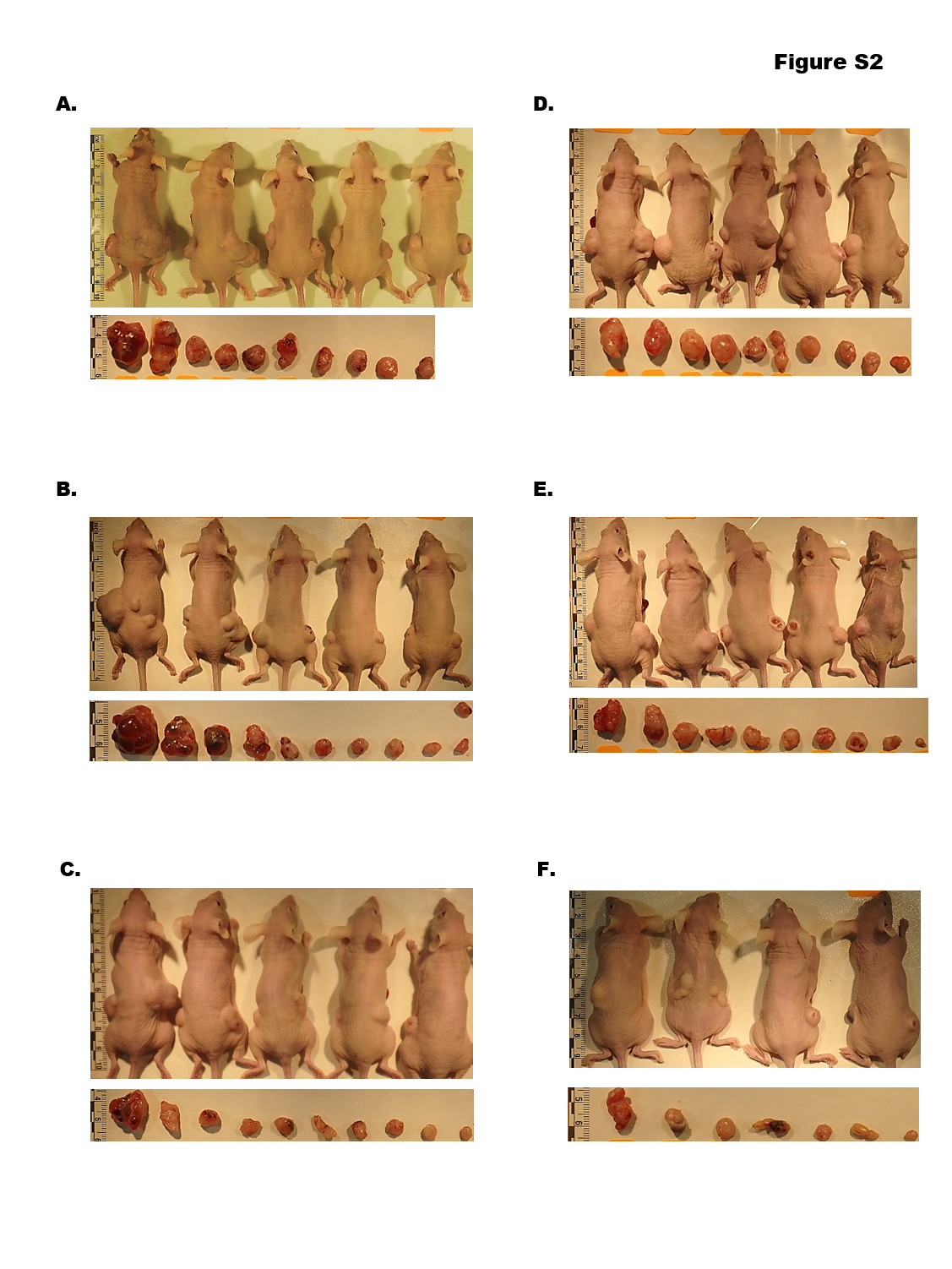

Supplement: Supplemental Material [file IDRD_A_1912209_SM7542.zip › Figure S2.tif]
